# Long-term effectiveness of ACE inhibitors or angiotensin receptor blockers in myocardial infarction with preserved left ventricular ejection fraction

**DOI:** 10.1093/ehjcvp/pvaf051

**Published:** 2025-08-31

**Authors:** Anna B C Humphreys, Bertil Lindahl, Anita Berglund, Vanessa Voelskow, Si Fang, Ole Fröbert, Robin Hofmann, Tomas Jernberg, Miguel A Hernán, Anthony A Matthews

**Affiliations:** Unit of Epidemiology, Institute of Environmental Medicine, Karolinska Institutet, 17177 Stockholm, Sweden; Department of Medical Sciences, Uppsala University, 751 85 Uppsala, Sweden; Unit of Epidemiology, Institute of Environmental Medicine, Karolinska Institutet, 17177 Stockholm, Sweden; Institute of Public Health, Charité—Universitätsmedizin Berlin, 10117 Berlin, Germany; MRC Integrative Epidemiology Unit, Population Health Sciences, Bristol Medical School, University of Bristol, Bristol BS8 2BN, UK; Department of Cardiology, Faculty of Health, Örebro University Hospital, 70362 Örebro, Sweden; Department of Clinical Medicine, Aarhus University Hospital, 8200 Aarhus, Denmark; Department of Clinical Pharmacology, Aarhus University Hospital, 8200 Aarhus, Denmark; Steno Diabetes Center Aarhus, Aarhus University Hospital, 8200 Aarhus, Denmark; Department of Clinical Science and Education, Division of Cardiology, Karolinska Institutet, Södersjukhuset, 11883 Stockholm, Sweden; Department of Clinical Sciences, Danderyd University Hospital-Karolinska Institutet, 18288 Stockholm, Sweden; Unit of Epidemiology, Institute of Environmental Medicine, Karolinska Institutet, 17177 Stockholm, Sweden; CAUSALab, Department of Epidemiology, Harvard T. H. Chan School of Public Health, Boston, MA 02115, USA; Department of Biostatistics, Harvard T. H. Chan School of Public Health, Boston, MA 02115, USA; Unit of Epidemiology, Institute of Environmental Medicine, Karolinska Institutet, 17177 Stockholm, Sweden

**Keywords:** Preserved left ventricular ejection fraction, Myocardial infarction, Target trial emulation, Observational study, Angiotensin-converting enzyme inhibitor, Angiotensin receptor blocker

## Abstract

**Aims:**

Angiotensin-converting enzyme inhibitors (ACEi) and angiotensin receptor blockers (ARB) are effective in the long-term treatment of myocardial infarction with reduced left ventricular ejection fraction (LVEF). However, it is unknown whether there is a benefit in myocardial infarction with preserved LVEF (≥50%).

**Methods and results:**

We used Swedish healthcare registries to emulate a target trial of ACEi/ARBs vs. no ACEi/ARBs for the prevention of a composite outcome (death, myocardial infarction, or heart failure) and its individual components among individuals under 75 years with myocardial infarction and LVEF ≥ 50% between September 2010 and June 2021. We estimated observational analogues of the intention-to-treat effect and the per-protocol effect with confounding adjustment via inverse probability weighting. The 10 697 individuals in the ACEi/ARB group were on average older (median 61 vs. 60 years) and more likely to be male (80.2% vs. 75.3% male) than the 4730 individuals in the no ACEi/ARB group. The estimated 5-year risk of the composite outcome was 7.8% (95% confidence interval 7.1%, 8.5%) in the ACEi/ARB group and 8.1% (7.0%, 9.3%) in the no ACEi/ARB group; risk difference −0.3% (−1.6%, 1.0%). After adjustment for adherence, the risk of the composite outcome was 6.5% (5.9%, 7.2%) in the ACEi/ARB group and 6.7% (5.6%, 8.1%) in the no ACEi/ARB group; risk difference −0.2% (−1.7%, 1.0%).

**Conclusion:**

The estimated risk of a composite of death, myocardial infarction or heart failure was similar in recipients and non-recipients of ACEi/ARB. Our estimates suggest ACEi/ARB treatment in myocardial infarction with preserved LVEF does not confer a benefit.

## Graphical Abstract

**Graphical Abstract:**
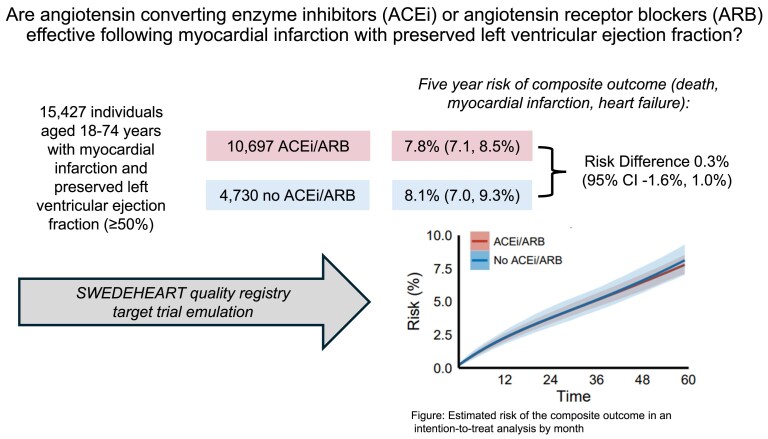

## Introduction

Angiotensin-converting enzyme inhibitors (ACEi) and angiotensin receptor blockers (ARB) are effective and recommended therapies following myocardial infarction with reduced left ventricular ejection fraction (LVEF), symptoms of heart failure, chronic kidney disease, hypertension, and/or diabetes.^1–6^ Indeed, the use of these drugs is a quality indicator for individuals with reduced LVEF.^7^ There is, however, little knowledge on the effectiveness of ACEi/ARBs in those with myocardial infarction with preserved LVEF (≥50%).^8^

Guidelines from both the European Society of Cardiology and the American Heart Association suggest a Class IIa or lower recommendation for ACEi/ARBs in all individuals with myocardial infarction regardless of LVEF.^8–10^ However, the few trials that assessed ACEi/ARBs in all individuals with myocardial infarction, irrespective of left ventricular fraction, estimated little or no effect, and are from an era before the widespread use of high-sensitive troponins, percutaneous coronary intervention, anti-thrombotic agents, and high-intensity statins.^11–15^ Observational studies provide conflicting evidence.^16–20^

To the best of our knowledge, no randomized trials are planned to evaluate the effect of ACEi/ARBs vs. no treatment on the risk of death, reinfarction, and heart failure following myocardial infarction with preserved LVEF and no indications for these drugs. The next best solution is, therefore, to use observational data. We designed a hypothetical pragmatic randomized trial, the target trial, that would answer this question. We then emulated the target trial using observational data from the Swedish Web-system for Enhancement and Development of Evidence-based care in Heart disease Evaluated According to Recommended Therapies (SWEDEHEART) registry and linked population registries.^21^

## Methods

A target trial emulation requires two steps: (i) specifying the protocol of the target trial, and (ii) emulating the target trial in the available observational data.^22^

### Specification of the target trial

The protocol of the target trial is outlined in *Table 1* and summarized herein. Eligible individuals would be 18–74 years, within 30 days of a hospital admission for a Type 1 myocardial infarction with preserved left ventricular systolic ejection fraction (≥50%) in any Swedish hospital that provides acute coronary care from 1 September 2010 to 30 June 2021; would have received standard myocardial infarction care (coronary angiography, anti-thrombotics, and statins) but no ACEi/ARBs; would have no indications (hypertension, heart failure, diabetes mellitus, or chronic kidney disease) for ACEi/ARBs, no contraindication to ACEi/ARBs (renal artery stenosis, hypotension), and no metastatic cancer or dementia within the last 3 years; would have an estimated glomerular filtration rate > 15 mL/min/1.73 m^2^ at their first test during their hospital admission; and would not be unemployed or on sick leave (see Discussion for full explanation).

**Table 1:** Outline of protocols of the target trial, and of an emulation of the target trial using the SWEDEHEART registry

Protocol component	Target trial	Target trial emulation using SWEDEHEART
Eligibility criteria^a^	· Day 1–30 after hospitalization for myocardial infarction as defined by the universal definition of MI, Type 1 · Age ≥ 18 years and age <75 years · Recruitment period 1 September 2010 until 30 June 2021 across all Swedish hospitals · Preserved left ventricular ejection fraction confirmed by echocardiography showing an ejection fraction ≥50% · Coronary angiography and on statins and anti-thrombotics at baseline · In employment, retired or studying · No contraindications to ACE-I or ARBs defined as poor kidney function (estimated glomerular filtration rate < 15 mL/min/1.73m^2^) at baseline, or hypotension, or renal artery stenosis in the previous 3 years · No indication for ACE-I/ARB other than for secondary prevention, defined as no ACE-I/ARB use, hypertension, chronic kidney disease, diabetes mellitus, or heart failure in the previous 3 years · No conditions that may influence the ability to comply, defined as dementia in the previous 3 years · No metastatic cancer in the previous 3 years	Same as the target trial, apart from: · All diagnoses were identified as primary or secondary diagnoses from the inpatient or outpatient sub registers of the National Patient Register. Hypertension, diabetes, and heart failure additionally identified from the SWEDEHEART registry · Previous ACE-I/ARB use identified from the Prescribed Drug Register or from a record of ACE-I/ARB on admission in the SWEDEHEART registry · Estimated glomerular filtration rate derived from the first creatinine measurement in SWEDEHEART · Statin and anti-thrombotic agent use identified from the Prescribed Drug Register
Treatment strategies	(1) ACEi or ARB throughout follow up unless a contraindication arises, defined as hypotension or renal artery stenosis (2) No ACEi or ARB throughout follow up unless indicated for reasons other than secondary prevention following myocardial infarction, defined as hypertension (and heart failure for the secondary outcomes of death and myocardial infarction)	Same as target trial. · If no record exists in National Prescribed Drug Register then the SWEDEHEART register was used which does not contain specific dates of drug prescription, but indicates if ACEi or ARB were given at discharge. In this case, prescription date was set at the date of discharge · Discontinuation dates of ACEi or ARB calculated based on the number of pills for that dispensation, and assuming a fixed dosage of one tablet per day. Treatment was considered continuous if there was a gap of <90 days between successive dispensations · All contraindications and indications identified as primary or secondary diagnosis in either the inpatient or outpatient sub registers of the National Patient Register after baseline
Treatment assignment	Individuals are randomly assigned to a treatment strategy at baseline and are aware of the strategy that they have been assigned to	We assumed that assignment (conceptualized as prescription) was as if randomized within levels of the baseline covariates in Supplementary material online, *Table S2*
Outcomes	Primary outcome: death, myocardial infarction, or heart failure Secondary outcomes: any individual component of the primary outcome	Primary outcome: death identified from the National Total Population Register, myocardial infarction from the SWEDEHEART registry, and heart failure from the National Patient Register (primary diagnosis)
Follow-up	Starts at baseline and ends at date of first outcome (separately for analysis of each secondary outcome), end of follow up (31 December 2022), or 5 years after baseline	Same as target trial
Estimands	Intention-to-treat and per protocol effects	Observational analogue of the intention-to-treat and per protocol effects
Statistical analysis	· Absolute risks in each group estimated using a pooled logistic regression model for the outcome with an indicator for assigned strategy, time of follow up, and a product term between follow up and assigned strategy. Covariates unbalanced at baseline used to estimate inverse probability of treatment weights. Risk differences then estimated · Per protocol analysis is the same except that individuals are censored when they deviate from their assigned treatment strategy. Time-varying inverse probability weights estimated to adjust prognostic factors associated with non-adherence · Non-parametric bootstrapping with 500 samples used to calculate 95% confidence intervals · Subgroup analysis by age, sex, and STEMI/NSTEMI	Same as target trial apart from: · All baseline covariates included in estimation of baseline inverse probability treatment weights

^a^All ICD-10 and ATC codes used to operationalize diagnoses and treatments in Supplementary material online, *Table S1*.

Eligible individuals would be randomly assigned to either ACEi/ARB or no ACEi/ARB and would be aware of the strategy they are assigned to. For those assigned to ACEi/ARB, the prescribing physician would be encouraged to aim for the maximum tolerated dose, and participants would be encouraged to continue treatment unless a contraindication develops (hypotension or renal artery stenosis). Individuals would be permitted to change drug class between ACEi and ARB. Those assigned to no ACEi/ARB would be encouraged to refrain from taking treatment unless an indication other than secondary prevention develops (hypertension for all outcomes, additionally heart failure for the individual death and myocardial infarction outcomes).

The outcomes of interest would be a composite of death from any cause, myocardial infarction, or heart failure, and the individual components of the composite outcome. Follow-up would start at assignment (baseline) and would end at the earliest of the outcome of interest, 31 December 2022, or 5 years after baseline. The causal contrasts of interest would be the intention-to-treat effect (effect of assignment to treatment) and the per-protocol effect (effect of treatment under full adherence to the protocol), defined as total effects.^23^

### Statistical analysis

For the intention-to-treat analysis, 5-year absolute risks would be estimated either non-parametrically via the Kaplan–Meier estimator or parametrically via a pooled logistic regression model with an indicator of assigned strategy, time of follow-up (restricted cubic spline with knots at 6, 12, 24, and 48 months), and a product term between time of follow-up and the indicator of assigned strategy. Prognostic factors imbalanced at baseline would be adjusted via unstabilized inverse probability (IP) weighting, and absolute risks would be compared via ratios and differences. The per-protocol analysis would be the same, except individuals would be censored if and when they stop adhering to their assigned treatment strategy, and time-varying IP weights would be estimated to adjust for baseline and time-varying prognostic factors associated with non-adherence. Subgroup analyses would be conducted according to ST elevation myocardial infarction/non-ST elevation myocardial infarction, age (<60 and ≥60 years), and sex.

### Emulation of the target trial using SWEDEHEART

We emulated the target trial in the SWEDEHEART quality registry, with linkage via the unique Swedish personal identifier to the Total Population Register, the National Patient Register, the National Prescribed Drug Register, and the Longitudinal Integrated Database for Health Insurance and Labour Market Studies (LISA).^21,24–26^ SWEDEHEART contains all individuals hospitalized for either acute coronary syndrome or undergoing coronary or valvular intervention in Sweden. The National Patient Register contains information on all inpatient admissions and outpatient consultations, whilst the National Prescribed Drug Register contains data on all dispensed medications. Data on country of birth, education, civil status, and income were obtained from LISA. The outcome of death was identified from the Total Population Register, myocardial infarction from the SWEDEHEART registry, and heart failure from the National Patient Register (limited to primary diagnoses).

The mapping of each component of the target trial protocol to the observational data is described below and in *Table 1* and Supplementary material online, *Table S1*. Treatment assignment was defined as prescription of an ACEi (Anatomic Therapeutic Chemical [ATC] codes starting with C09A, and C09B) or ARB (ATC codes starting with C09C and C09D) in the Prescribed Drug Register, or a record of either medication on discharge in SWEDEHEART, within 30 days of hospital admission. Dosage information was not available, and the intended length of each dispensation in the ACEi/ARB group was therefore calculated by assuming participants were recommended to take one pill per day; with adherence then defined as a gap of <90 days between the end date of one dispensation and the following dispensation, unless this was preceded by a contraindication. In the no ACEi/ARB group, adherence was defined by continuing to not dispense ACEis or ARBs, unless dispensation was preceded by a new indication.

We assumed that treatment assignment was as if randomized within levels of the following baseline covariates: hospital, index year of admission, age, sex, birth country, civil status, income, education, employment status, smoking status, previous myocardial infarction, previous stroke, previous percutaneous coronary intervention, previous cardiac surgery, infarction type, cardiopulmonary resuscitation before hospital, thrombolysis before hospital, cardiogenic shock, ECG rhythm, ECG QRS annotation, ECG ST- and T-wave changes, percutaneous coronary intervention, intravenous beta-blockers, intravenous diuretics, intravenous inotropic drugs, intravenous nitrates, heart rate, systolic blood pressure, diastolic blood pressure, low-density lipoprotein cholesterol, high-density lipoprotein cholesterol, estimated glomerular filtration rate, body mass index, angiography finding, stenosis class, proportion stenosis, beta blockers, calcium channel blockers, diuretics, nitrates, and diabetes treatments (see Supplementary material online, *Table S2* for full variable definitions).

The intention-to-treat analysis was the same as the target trial with adjustment for all baseline prognostic factors listed above, and the per-protocol analysis was the same as for the target trial. The per-protocol analysis included adjustment for the baseline variables plus the following time-varying prognostic factors: diagnosis of renal disease; diagnosis of Type 1 or Type 2 diabetes, and dispensation of beta-blockers, calcium channel blockers, diuretics, nitrates, and diabetes treatment. Continuous variables with missing data were categorized to include a missing indicator (see Supplementary material online, *Table S2* for variable list), with categorical variables also including a missing category. Bootstrapping with 500 samples was used to calculate 95% confidence intervals.

### Sensitivity analyses

We conducted several sensitivity analyses to assess the robustness of the effect estimates. For the intention-to-treat analysis, we aimed to understand the impact of (i) the choice of eligibility criteria by excluding those without obstructive coronary artery disease; (ii) restricting anti-thrombotic agents to long-term agents; (iii) the COVID-19 pandemic by excluding those who were eligible after 2020; (iv) different missing data assumptions by (a) carrying out a complete case analysis and (b) imputing the median value for continuous variables; (v) modelling assumptions by including baseline covariates in the pooled logistic regression model instead of using IP weights; (vi) adjustment for measured covariates by including only age and sex as covariates; (vii) truncating inverse probability weights to the 99th percentile, and (viii) potential misalignment of eligibility, treatment assignment and the start of follow up (full details in the Supplementary material online, appendix). We also aimed to assess the impact of censoring at death for the outcomes of (i) myocardial infarction and (ii) heart failure. For the per-protocol analysis, we assessed the impact of (i) adjustment for measured covariates by adjusting for age and sex only; (ii) different methods to define continuous treatment by (a) increasing the time allowed between successive dispensations from 90 to 180 days to understand if having a larger gap between dispensations affected results and (b) assuming that each successive dispensation corresponded to 90 fixed days of treatment with a 90-day grace period. Finally, for the per-protocol analysis for the outcome of heart failure, we aimed to assess the impact of decreasing the time of the outcome by 6 months (full details in Supplementary material online, *Appendix*).

## Results

**Figure 1:**
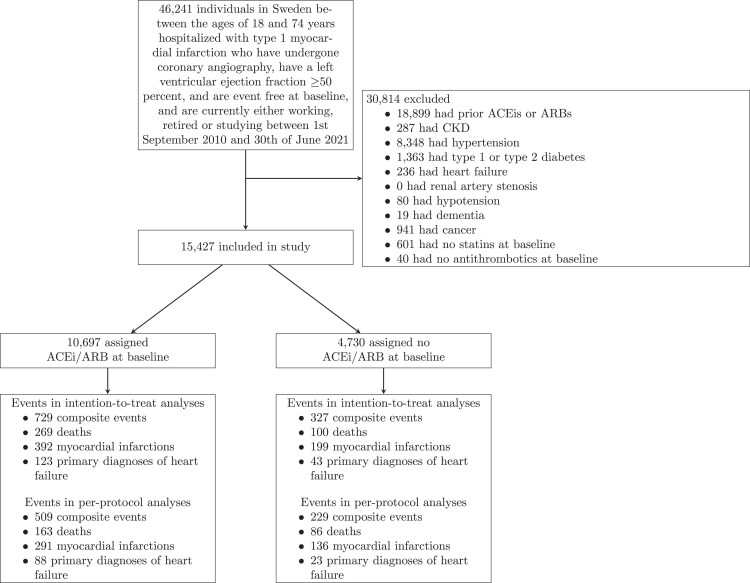
Flowchart for selection of eligible individuals into an emulation of a target trial of angiotensin-converting enzyme inhibitors and angiotensin receptor blockers vs. no angiotensin-converting enzyme inhibitors and angiotensin receptor blockers in individuals with myocardial infarction with preserved left ventricular ejection fraction.

*Figure 1* shows a flowchart of selection into the target trial emulation, resulting in 15 427 eligible individuals. *Table 2* presents their baseline characteristics: the 10 697 individuals in the ACEi/ARB group were on average older, more likely to be male, and less likely to have had a previous percutaneous coronary intervention than the 4730 individuals in the no ACEi/ARB group. Individuals in the ACEi/ARB group had higher blood pressure and were more likely to present with STEMI, be treated with beta blockers, and have a greater degree of coronary stenosis.

**Table 2:** Baseline characteristics of eligible individuals for an emulation of a target trial of ACEi/ARB vs. no ACEi/ARB in individuals with myocardial infarction and preserved left ventricular ejection fraction

	ACE I/ARB	No ACE I/ARB	Missing	SMD	SMD after IP weighting
	10697	4730			
Characteristics					
Age	61.0 [54.0, 68.0]	60.0 [52.0, 67.0]	0	0.139	0.003
Female	2123 (19.8)	1167 (24.7)	0	0.116	0.013
Country of birth			4.4	0.118	0.021
Sweden	8743 (85.4)	3748 (83.0)			
Other Nordic countries	404 (3.9)	142 (3.1)			
Europe excluding Nordics	335 (3.3)	146 (3.2)			
Outside of Europe	754 (7.4)	477 (10.6)			
Civil status			0.4	0.026	0.003
Married/partner	6118 (57.5)	2769 (58.7)			
Unmarried/not partnered	2183 (20.5)	933 (19.8)			
Divorced	2010 (18.9)	865 (18.3)			
Widow/widower/surviving partner	337 (3.2)	147 (3.1)			
Occupational status			0	0.096	0.004
Working	6554 (61.3)	3101 (65.6)			
Retired	4034 (37.7)	1569 (33.2)			
Student	109 (1.0)	60 (1.3)			
Yearly disposable income (Per 100 kronor)	2499.0 [1685.0, 3378.0]	2623.0 [1742.2, 3550.8]	0.4	0.065	0.011
Educational level			1.1	0.111	0.019
Pre-secondary education less than 9 years old	1155 (10.9)	452 (9.7)			
Pre-secondary education of 9 years (equivalent)	1372 (13.0)	574 (12.3)			
Secondary education up to 2 years	3634 (34.3)	1478 (31.6)			
Secondary education of 3 years	1531 (14.5)	673 (14.4)			
Post-secondary education less than 3 years old	1319 (12.5)	653 (14.0)			
Post-secondary education 3 years or more	1569 (14.8)	845 (18.1)			
Smoking status			1.2	0.069	0.018
Never smoker	3869 (36.6)	1862 (39.9)			
Ex-smoker (>1 month)	3279 (31.0)	1369 (29.4)			
Smoker	3435 (32.5)	1432 (30.7)			
Previous myocardial infarction	324 (3.0)	223 (4.7)	0.1	0.088	0.003
Previous stroke	79 (0.7)	47 (1.0)	0.1	0.028	0.008
Previous percutaneous coronary intervention	259 (2.4)	200 (4.2)	0.1	0.101	0.004
Cardiac surgery	62 (0.6)	50 (1.1)	0.1	0.053	0.009
Presentation					
NSTEMI	5836 (54.6)	3408 (72.1)	0	0.369	0.048
Cardiopulmonary resuscitation	216 (2.0)	47 (1.0)	1	0.084	0.018
Thrombolysis	59 (0.6)	27 (0.6)	0.3	0.003	0.006
Cardiogenic shock	45 (0.4)	18 (0.4)	0.4	0.006	0.013
ECG rhythm			0.4	0.055	0.013
Sinus	10410 (97.7)	4602 (97.7)			
Ability flicker/flutter	140 (1.3)	79 (1.7)			
Other	107 (1.0)	28 (0.6)			
ECG QRS annotation			1	0.086	0.025
Normal	9021 (85.1)	4075 (87.1)			
Pacemaker	18 (0.2)	5 (0.1)			
Left block branch	65 (0.6)	26 (0.6)			
Pathological Q-wave	629 (5.9)	209 (4.5)			
Right block branch	214 (2.0)	117 (2.5)			
Other	648 (6.1)	245 (5.2)			
ECG ST- and T- wave changes			0.6	0.39	0.052
Normal	2666 (25.0)	1818 (38.7)			
ST elevation	4688 (44.0)	1278 (27.2)			
ST depression	1771 (16.6)	760 (16.2)			
Pathological T-wave	1007 (9.5)	529 (11.3)			
Other	514 (4.8)	310 (6.6)			
Measurements					
Heart rate	72.0 [62.0, 84.0]	71.0 [62.0, 82.0]	0.3	0.049	0.001
Systolic blood pressure (mm/Hg)	150.0 [134.0, 169.0]	143.0 [128.0, 160.0]	0.3	0.3	0.055
Diastolic blood pressure (mm/Hg)	90.0 [80.0, 100.0]	85.0 [76.0, 95.0]	3.8	0.258	0.043
HDL cholesterol (mmol/L)	1.2 [1.0, 1.4]	1.2 [1.0, 1.4]	10.4	0.048	0.009
LDL cholesterol (mmol/L)	3.5 [2.8, 4.2]	3.5 [2.8, 4.2]	11	0.022	0.003
Estimated Glomerular Filtration Rate	90.0 [79.6, 97.9]	90.4 [79.3, 98.5]	2.3	0.017	0.024
Body mass index (kg/m^2^)	27.0 [25.0, 30.0]	27.0 [24.0, 29.0]	3	0.156	0.024

SMD, standardized mean difference; IP weighting, inverse probability weighting; NSTEMI, non-ST elevation myocardial infarction; ECG, electrocardiogram; HDL, high-density lipoprotein; LDL, low-density lipoprotein.

### Intention-to-treat analysis

**Figure 2:**
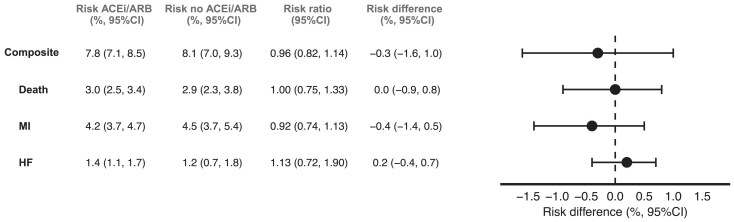
Table and forest plot showing estimated risks and risk differences for the intention-to-treat effect of the composite outcome, death, myocardial infarction and heart failure by 5 years in an emulation of a target trial of angiotensin-converting enzyme inhibitors and angiotensin receptor blockers vs. no angiotensin-converting enzyme inhibitors and angiotensin receptor blockers.

**Figure 3:**
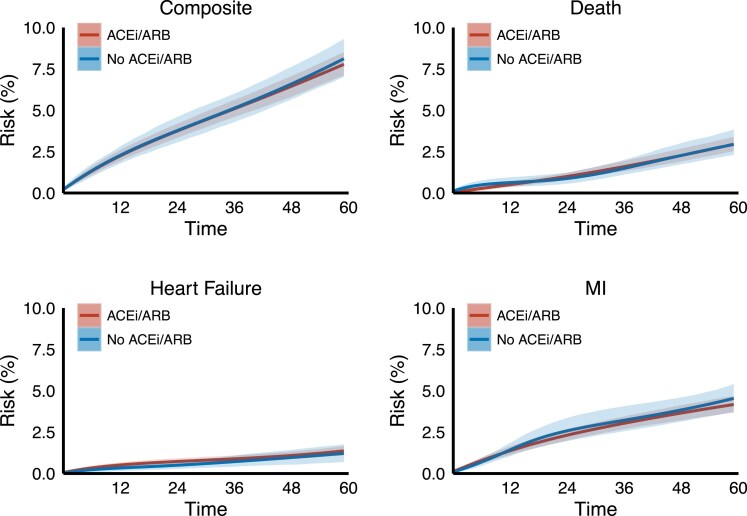
Estimated risk of the composite outcome, death, myocardial infarction and heart failure in those assigned angiotensin-converting enzyme inhibitors and angiotensin receptor blockers vs. no angiotensin-converting enzyme inhibitors and angiotensin receptor blockers in the intention-to-treat analysis (shaded intervals represent limits of the pointwise 95% CIs).

Median follow-up was 60 months in both groups. After adjustment for all baseline covariates, the estimated 5-year risk of the composite outcome was 7.8% (95% confidence interval 7.1%, 8.5%) in the ACEi/ARB group and 8.1% (7.0%, 9.3%) in the no ACEi/ARB group, corresponding to a risk difference of −0.3% (−1.6%, 1.0%) and a risk ratio of 0.96 (0.82, 1.14) (*Figures 2* and *3*). For the individual components of the composite outcome, the estimated 5-year risk differences were 0.0% (−0.9%, 0.8%) for death, −0.4% (−1.4%, 0.5%) for myocardial infarction and 0.2% (−0.4%, 0.7%) for heart failure. Results of the subgroup analyses were broadly similar to the main analysis (see Supplementary material online, *Table S4*).

### Per protocol analysis

**Figure 4:**
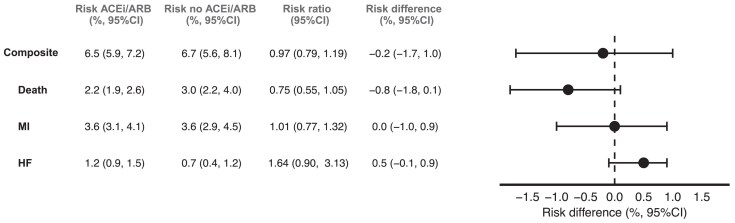
Table and forest plot showing estimated risks and risk differences for the per-protocol effect of the composite outcome, death, myocardial infarction, and heart failure by 5 years in an emulation of a target trial of angiotensin-converting enzyme inhibitors and angiotensin receptor blockers vs. no angiotensin-converting enzyme inhibitors and angiotensin receptor blockers.

At 5 years, adherence was 45.6% in the ACEi/ARB group and 79.1% in the no ACEi/ARB group. After censoring at non-adherence, median follow-up was 45 months in the ACEi/ARB group, and 58 months in the no ACEi/ARB group. After adjusting for baseline and time-varying covariates, the estimated 5-year risk of the composite outcome was 6.5% (5.9%, 7.2%) in the ACEi/ARB group and 6.7% (5.6%, 8.1%) in the no ACEi/ARB, corresponding to a risk difference of −0.2% (−1.7%, 1.0%) and a risk ratio of 0.97 (0.79, 1.19), as seen in *Figure 4* and Supplementary material online, *Figure S1*. For the individual components of the composite outcome, the estimated 5-year risk differences were −0.8% (−1.8%, 0.1%) for death, 0.0% (−1.0%, 0.9%) for myocardial infarction, and 0.5% (−0.1%, 0.9%) for heart failure.

### Sensitivity analyses

Results from all intention-to-treat and per-protocol sensitivity analyses were broadly similar to the main analysis (see Supplementary material online, *Tables S5*  through  *S8*).

## Discussion

We found effect estimates close to null, with 95% confidence intervals that are compatible with both beneficial and harmful effects for ACEi/ARBs on the 5-year risk of death, myocardial infarction, and heart failure in low-risk individuals presenting with an acute myocardial infarction with preserved LVEF.

While ACEi/ARB are well known to be beneficial in individuals with myocardial infarction and reduced LVEF,^1–4^ no modern randomized trials have been conducted among those with preserved ejection fraction.^11–15^ Therefore, direct comparisons of our estimates with existing literature are challenging. Existing observational studies differed in eligibility criteria, requirement for active follow up, modelling strategies, time zero, and follow-up duration, which also makes comparisons difficult.^16–18^

The comprehensive data within SWEDEHEART and linked registers include extensive information on treatments, baseline confounders, and outcomes. The use of the target trial emulation framework helps to overcome common biases arising in observational studies such as the misalignment of time zero, when eligibility must be assessed, treatment assigned and follow up started.^22^

Our study, however, has several limitations. First, as in any observational study, some residual confounding may remain. For example, individuals in the ACEi/ARB group had a higher baseline blood pressure than those in the no ACEi/ARB group despite excluding individuals with previous use of these drugs or a diagnosis of hypertension. However, the magnitude of residual confounding should be limited as the difference in systolic and diastolic blood pressure between the two groups did not exceed 7 mmHg. Second, there were limited data on prognostic factors after baseline (e.g. frailty, clinical symptoms), which may have prevented us from full confounding adjustment in the per protocol analysis. However, we restricted the eligible population to individuals younger than 75 years who received standard care for myocardial infarction, were employed, studying or retired and did not have a diagnosis of metastatic cancer in the last 3 years. This restriction decreases the probability of substantial confounding at the expense of potentially limited transportability of the results to older, more frail individuals.^27^ Third, data on several contraindications for the study drugs were not available in the data (intolerance to ACEi/ARBs, end stage renal failure, and hyperkalaemia). However, the prevalence of these conditions in the study population is expected to be low. Finally, we did not have access to information on dosing nor primary care data, which would give us more detailed information on routine biomarker measurement and diagnosis of less severe diseases that do not require specialized care. However, a sensitivity analysis that measured adherence by a fixed 3-month dispensation length (the common Swedish practice for this treatment) did not meaningfully change results.

In summary, we estimated that ACEi/ARBs had little or no effect on the 5-year risk of death, myocardial infarction, and heart failure among low-risk individuals with an acute myocardial infarction and preserved LVEFs. Our results do not provide evidence to support the recommendation to use ACE inhibitors routinely in the treatment of low-risk individuals under the age of 75 years with myocardial infarction and preserved LVEF.

## Supplementary Material

pvaf051_Supplementary_Data

## Data Availability

In agreement with Swedish laws and regulations, only researchers who fulfil legal requirements may access personal sensitive data. For further information, contact the corresponding author. All analysis codes are available at: https://github.com/Annahum/tte_acei.
