# Supplementary material for: Long-term effectiveness of ACE inhibitors or angiotensin receptor blockers in myocardial infarction with preserved left ventricular ejection fraction
Source: Eur Heart J Cardiovasc Pharmacother. 2025 Aug 31;11(7):600–9. doi: 10.1093/ehjcvp/pvaf051 (PMC12582658; doi:10.1093/ehjcvp/pvaf051)
Supplement: pvaf051_Supplementary_Data [file pvaf051_supplementary_data.docx]

# Supplementary appendix

Contents

[Supplementary appendix 1](#_Toc206496002)

[Sensitivity analysis to evaluate potential bias arising from the misalignment of eligibility, treatment assignment, and start of follow up 2](#_Toc206496003)

[Sensitivity analysis to evaluate potential bias arising in the per protocol analysis for heart failure 4](#_Toc206496004)

[Supplementary Table 1 – ICD-10 and ATC codes used to define diagnoses and treatments for eligibility criteria, treatment strategies and outcomes 5](#_Toc206496005)

[Supplementary Table 2 – covariate definitions 6](#_Toc206496006)

[Supplementary Table 3: Baseline characteristics of eligible individuals for an emulation of a target trial of ACEi/ARB versus no ACEi/ARB in individuals with myocardial infarction and preserved left ventricular ejection fraction, continued from Table 2 11](#_Toc206496007)

[Supplementary Table 4 - Estimated 5-Year risks, and risk differences for the composite outcome for the intention-to-treat effect, stratified by subgroup 13](#_Toc206496008)

[Supplementary Table 5 - Sensitivity analyses for intention-to-treat analysis with composite outcome 14](#_Toc206496009)

[Supplementary Table 6 - Sensitivity analyses for intention-to-treat analysis with heart failure and myocardial infarction outcomes 15](#_Toc206496010)

[Supplementary Table 7 - Sensitivity analyses for per-protocol analysis with composite outcome 16](#_Toc206496011)

[Supplementary Table 8 - Sensitivity analyses for per-protocol analysis with heart failure outcome 17](#_Toc206496012)

[Supplementary Figure 1 - Estimated risk of the composite outcome, death, myocardial infarction (MI) and heart failure (HF) and in those assigned ACEi/ARB versus no ACEi/ARB in the per-protocol analysis^*^ 18](#_Toc206496013)

### Sensitivity analysis to evaluate potential bias arising from the misalignment of eligibility, treatment assignment, and start of follow up

Time is measured in 30-day increments in our target trial emulation. This time scale, as opposed to more granular follow up (e.g. daily), is a simplified approach that reduces the computational processing time for long term outcomes that are measured up to five years later. Accordingly, baseline (time zero) is considered the first 30 days after the initial hospital admission for the index myocardial infarction. At baseline, there are three processes that must occur: the application of eligibility criteria, treatment assignment, and the start of follow up. The use of a baseline 30 day period can, however, lead to two distinct biases resulting from misalignment of these three processes (1).

First, the application of eligibility criteria may not occur at the same time as treatment assignment. For individuals in the no ACE inhibitor/ARB group, treatment assignment is defined by the absence of a relevant prescription or record in the SWEDEHEART register, and accordingly their assignment can only be assessed at the end of the 30 day period, likely after these individuals became eligible. An alternative situation can arise where an individual fulfils eligibility criteria after they have been assigned treatment. This individual would not be eligible for our target trial (where an individual would need to fulfil eligibility criteria before they can be assigned treatment), yet would be included in our main analysis. For example, an individual included in our target trial emulation may be treated with an ACE inhibitor 6 days after their hospital admission but may not receive a statin until day 10.

Second, individuals by definition cannot have an event at baseline. The use of a 30 day period thus can introduce immortal time as individuals must be event free (i.e. not have a recurrent myocardial infarction, diagnosis of heart failure or die) at baseline, which is up to 30 days after their hospital admission in our analysis.

We therefore conducted a sensitivity analysis to explore the potential impact of a misalignment of these three processes. We first identified the day within the 30 days of their hospital admission in which an individual fulfilled the eligibility criteria, and allowed a 30 day grace period after initial admission for myocardial infarction to receive treatment. The treatment an individual would be assigned was not necessarily known on day zero. Accordingly, we created a clone of each individual the day they became eligible with one clone assigned to the ACE inhibitor/ ARB group and the other to the no ACE inhibitor/ARB group. Immortal time was prevented by following individuals from day zero. Clones assigned to the no ACE inhibitor/ARB group were censored the day in which they received an ACE inhibitor/ARB prescription and those assigned to the ACE inhibitor/ARB group would be censored at the end of 30 days if they had not received a prescription by that point.

1. Matthews, A.A., Dahebreh, I.J., MacDonald, C.J. *et al.* Prospective benchmarking of an observational analysis in the SWEDEHEART registry against the REDUCE-AMI randomized trial. *Eur J Epidemiol* **39**, 349–361 (2024). https://doi.org/10.1007/s10654-024-01119-3

### Sensitivity analysis to evaluate potential bias arising in the per protocol analysis for heart failure

ACE inhibitors and angiotensin receptor blockers are commonly used in the treatment of heart failure. In the per protocol analysis, individuals in the no ACEi/ARB group may be censored due to starting treatment for heart failure when symptomatic, prior to being formally diagnosed. It is possible therefore that there is residual confounding as time varying information on symptoms was not available. We explored the potential impact of individuals receiving treatment prior to diagnosis by artificially reducing the time to heart failure diagnosis by six months (and if the heart failure diagnosis was originally under six months, set to month one).

### Supplementary Table 1 – ICD-10 and ATC codes used to define diagnoses and treatments for eligibility criteria, treatment strategies and outcomes

| **Diagnoses** | **ICD-10 codes** |
| --- | --- |
| Hypotension | I95 |
| Renal artery stenosis | I701 |
| Dementia | G30, G31, G32 |
| Heart failure | I50 |
| Hypertension | I10, I15 |
| Diabetes | E10, E11, E12, E13, E14 |
| Chronic kidney disease | N17, N18, N19 |
| Cancer | C00 - C97 |
| **Treatments** | **ATC codes** |
| ACE inhibitor | C09A, C09B |
| Angiotensin II Receptor Blocker | C09C, C09D |
| Statins | C10AA |
| Antithrombotic treatments | B01 |

### Supplementary Table 2 – covariate definitions

| **Covariate** | **Register** | **Definition** | **Values** | **Form** | **Baseline/time-updated** |
| --- | --- | --- | --- | --- | --- |
| **Hospital** | RIKSHIA (SWEDEHEART) | Hospital where individual was admitted |  | Categorical | Baseline |
| **Year of index** | RIKSHIA (SWEDEHEART) | Year an individual has their index date | 2010-2021 | Categorical | Baseline |
| **Age** | RISKHIA  (SWEDEHEART) | Age at hospital admission |  | Restricted cubic spline with five knots | Baseline |
| **Sex** | RIKSHIA (SWEDEHEART) | Sex | 1 = Male \| 2 = Female | Categorical | Baseline |
| **Birth country** | LISA | Country of birth | 1 = Sweden \| 2 = Other Nordic country \| 3 = Europe excluding Nordic countries \| 4 = Outside of Europe \| 9 = Unknown | Categorical | Baseline |
| **Civil Status** | LISA | Civil status | 1 = Married/ partner \| 2 = Unmarried/ not partnered \| 3 = Divorced \| 4 =  Widow/ widower/ surviving partner \| 9 = Unknown | Categorical | Baseline |
| **Income** | LISA | Disposable income - total income received minus taxes paid per 100 SEK | <1000 \| 1001 - 2000 \| 2001 - 3000 \|3001 - 4000 \| 4001+ \| NA | Categorical  Sensitivity: Restricted cubic spline with five knots, with the median imputed for missing data | Baseline |
| **Education** | LISA | Highest level of education received | 1 = Pre-secondary education less than 9 years old \| 2 = Pre-secondary education of 9 years (equivalent) \| 3 = Secondary education up to 2 years \| 4 = Secondary education of 3 years \| 5 = Post-secondary education less than 3 years old \| 6 = Post-secondary education 3 years or more or postgraduate education \| 9 = Unknown | Categorical | Baseline |
| **Employment status** | RIKSHIA (SWEDEHEART) | Self reported employment | 1 = Working \| 2 = Sick Leave \| 3 = Unemployed \| 4 = Retired\| 5 = Student \| 9 = Unknown | Categorical | Baseline |
| **Smoking status** | RIKSHIA (SWEDEHEART) | Self reported smoking at admission (also applies to hookah) | 0 = Never smoker \| 1 = Ex smoker> 1 month \| 2 = Smoker \| 9 = Unknown | Categorical | Baseline |
| **Previous myocardial infarction** | RIKSHIA (SWEDEHEART) | Diagnosis of myocardial infarction as any time before admission, either through documentation in patient record or self report. Also specify if had silent myocardial infarction that the patient is unaware of, but which has been shown on an ECG. | 0 = No \| 1 = Yes \| 9 = Unknown | Categorical | Baseline |
| **Previous stroke** | RIKSHIA (SWEDEHEART) | Diagnosis of ischemic stroke or bleeding at any time before admission, not including TIA, through documentation in patient record | 0 = No \| 1 = Yes \| 9 = Unknown | Categorical | Baseline |
| **Previous percutaneous coronary intervention** | RIKSHIA (SWEDEHEART) | Specify whether the patient has previously undergone a PCI (Percutaneous Coronary Intervention) of any type before the current insertion (balloon dilation, atherectomy, stent or other). Retrieved from previous registration | 0 = No \| 1 = Yes \| 9 = Unknown | Categorical | Baseline |
| **Previous cardiac surgery** | RIKSHIA (SWEDEHEART) | Specify whether the patient has undergone open heart surgery before this hospitalization | 0 = No \| 1 = Yes \| 9 = Unknown | Categorical | Baseline |
| **Infarction type** | RIKSHIA (SWEDEHEART) | Whether the patient is perceived by the treating physician as a STEMI or non-STEMI patient | 1 = STEMI \| 2 = NSTEMI | Categorical | Baseline |
| **Cardiopulmonary resuscitation before hospital** | RIKSHIA (SWEDEHEART) | Specify whether CPR (even defibrillation only) was performed prior to arrival at hospital | 0 = No \| 1 = Yes \| 9 = Unknown | Categorical | Baseline |
| **Thrombolysis before hospital** | RIKSHIA (SWEDEHEART) | Specify whether thrombolysis was given prior to arrival in hospital | 0 = No \| 1 = Yes \| 9 = Unknown | Categorical | Baseline |
| **Cardiogenic shock** | RIKSHIA (SWEDEHEART) | Cardiogenic shock on arrival to hospital | 0 = No \| 1 = Yes \| 9 = Unknown | Categorical | Baseline |
| **ECG rhythm** | RIKSHIA (SWEDEHEART) | ECG rhythm with regards to the ECG that is the basis for decision making for admission | 1 = Sinus \| 2 = Atrial flicker / flutter \| 8 = Other \| 9 = Unknown | Categorical | Baseline |
| **ECG QRS annotation** | RIKSHIA (SWEDEHEART) | QRS annotation with regards to the ECG that is the basis for decision making for admission | 1 = Normal \| 2 = Pacemaker \| 3 = Left branch block \| 4 = Pathological Q wave \| 5 = Right branch block \| 8 = Other \| 9 = Unknown | Categorical | Baseline |
| **ECG ST- & T-wave changes** | RIKSHIA (SWEDEHEART) | ST- & T-wave changes with regards to the ECG that is the basis for decision making for admission | 1 = Normal \| 2 = ST elevation \| 3 = ST depression \| 4 = Pathological T-wave \| 8 = Other \| 9 = Unknown | Categorical | Baseline |
| **Percutaneous coronary intervention** | RIKSHIA (SWEDEHEART) | Specify whether the patient underwent percutaneous coronary intervention | 0 = No \| 1 = Yes \| 9 = Unknown | Categorical | Baseline |
| **IV beta blockers** | RIKSHIA (SWEDEHEART) | Specify whether intravenous beta blockers were given during the hospitalization, most recent instance during hospitalization | 0 = No \| 1 = Yes \| 9 = Unknown | Categorical | Baseline |
| **IV diuretics** | RIKSHIA (SWEDEHEART) | Specify whether intravenous diuretics were given during the hospitalization, most recent instance during hospitalization | 0 = No \| 1 = Yes \| 9 = Unknown | Categorical | Baseline |
| **IV inotropic drugs** | RIKSHIA (SWEDEHEART) | Specify whether intravenous inotropic drugs were given during the hospitalization, most recent instance during hospitalization | 0 = No \| 1 = Yes \| 9 = Unknown | Categorical | Baseline |
| **IV nitrates** | RIKSHIA (SWEDEHEART) | Specify whether intravenous nitrates were given during the hospitalization, most recent instance during hospitalization | 0 = No \| 1 = Yes \| 9 = Unknown | Categorical | Baseline |
| **Heart rate** | RIKSHIA (SWEDEHEART) | Heart rate (beats per minute). It should be the first heart rate noted by healthcare professionals. | <59 \| 60-99 \| 100+ \| NA | Main: Categorical  Sensitivity: Restricted cubic spline with five knots, with the median imputed for missing data | Baseline |
| **Systolic blood pressure** | RIKSHIA (SWEDEHEART) | Systolic blood pressure. It should be the first blood pressure noted by healthcare professionals, which includes primary care physicians / ambulance and emergency room personnel | <119\| 120-139 \| 140+ \| NA | Main: Categorical  Sensitivity: Restricted cubic spline with five knots, with the median imputed for missing data | Baseline |
| **Diastolic blood pressure** | RIKSHIA (SWEDEHEART) | Diastolic blood pressure. It should be the first blood pressure noted by healthcare professionals, which includes primary care physicians / ambulance and emergency room personnel | <79 \| 80-89 \| 90+ \| NA | Main: Categorical  Sensitivity: Restricted cubic spline with five knots, with the median imputed for missing data | Baseline |
| **Low density lipoprotein cholesterol** | RIKSHIA (SWEDEHEART) | LDL in mmol/L is calculated primarily as total cholesterol - HDL - (0.45 x triglycerides) provided these values ​​are present and that the value of triglycerides is <= 4.5 mmol / L. If the calculation does not yield any results, the manually entered LDL value is used if available. Negative values ​​are set to Missing. | <3.3 \| 3.4-4.8 \| 4.9+ \| NA | Main: Categorical  Sensitivity: Restricted cubic spline with five knots, with the median imputed for missing data | Baseline |
| **High density lipoprotein cholesterol** | RIKSHIA (SWEDEHEART) | HDL in mmol/L, should be taken during first day of care of heart attack | <0.9 \| 1.0-1.5 \| 1.5+ \| NA | Main: Categorical  Sensitivity: Restricted cubic spline with five knots, with the median imputed for missing data | Baseline |
| **Estimated glomerular filtration rate*** | RIKSHIA (SWEDEHEART) | Creatinine, takes the first lab value (non missing) during admission. Estimated Glomerular Filtration Rate calculated using CKD EPI formula | <44 \| 45-59 \| 60-89 \| 90+ \| NA | Main: Categorical  Sensitivity: Restricted cubic spline with five knots, with the median imputed for missing data | Baseline |
| **Body mass index** | RIKSHIA (SWEDEHEART) | Body mass index based on weight and height at admission, mainly through asking patient (but could be measured) | <18.4 \| 18.5-24.9 \| 25.0-29.9 \| 30.0+ \| NA | Main: Categorical  Sensitivity: Restricted cubic spline with five knots, with the median imputed for missing data | Baseline |
| **Angiography finding** | SCAAR (SWEDEHEART) | Finding from angiography | 2 = 1 vessel not left main \| 3 = 2 vessels not left main \| 4 = 3 vessels not left main \| 5 = left main | Categorical | Baseline |
| **Stenosis class** | SCAAR (SWEDEHEART) | Stenosis according to J Am Coll Cardiol 1988: 12 (2): 529-45. | 1 = A \| 2 = B1 \| 3 = B2 \| 4 = C \| 5 = B1 Bifurcation \| 6 = B2 Bifurcation \| 7 = C Bifurcation \| 9 = Other | Categorical | Baseline |
| **Proportion stenosis** | SCAAR (SWEDEHEART) | Proportion stenosis in artery with highest level of stenosis (if more than one) | 1 = 0% \| 2 = <50% \| 3 = 50-69% \| 4 = 70-89% \| 5 = 90-99% \| 6 = 100% \| 9 = Unknown | Categorical | Baseline |
| **Renal disease** | National Patient Register | ICD-10 code (N17-19) in the inpatient or outpatient register as a primary or secondary diagnosis within after baseline. | 0 = No \| 1 = Yes | Categorical | Time updated |
| **Diabetes** | National Patient Register | ICD-10 code (E10, E11, E14 ) in the inpatient or outpatient register as a primary or secondary diagnosis after baseline. | 0 = No \| 1 = Yes | Categorical | Time updated |
| **Beta blockers** | RIKSHIA (SWEDEHEART)  Prescribed Drug Register | Regular beta blocker use prior to hospitalisation.  Dispensation ATC code (C07) in the prescribed drug register within 3 years prior | 0 = No \| 1 = Yes \| 9 = Unknown  0 = No \| 1 = Yes | Categorical | Baseline  Both |
| **Calcium channel blockers** | RIKSHIA (SWEDEHEART)  Prescribed Drug Register | Regular calcium channel blocker use prior to hospitalisation  Dispensation ATC code (C08) in the Prescribed Drug Register within 3 years prior | 0 = No \| 1 = Yes \| 9 = Unknown  0 = No \| 1 = Yes | Categorical | Baseline  Both |
| **Diuretics** | RIKSHIA (SWEDEHEART)  Prescribed Drug Register | Regular diuretic use prior to hospitalisation  Dispensation ATC code (C03) in the Prescribed Drug Register within 3 years prior | 0 = No \| 1 = Yes \| 9 = Unknown  0 = No \| 1 = Yes | Categorical | Baseline  Both |
| **Nitrates** | RIKSHIA (SWEDEHEART)  Prescribed Drug Register | Regular nitrate use prior to hospitalisation  Dispensation ATC code (C01DA) in the Prescribed Drug Register within 3 years prior | 0 = No \| 1 = Yes \| 9 = Unknown  0 = No \| 1 = Yes | Categorical | Baseline  Both |
| **Diabetes treatment** | RIKSHIA (SWEDEHEART)  Prescribed Drug Register | Regular diabetes treatment prior to hospitalisation  Dispensation ATC code (A10) in the Prescribed Drug Register within 3 years prior | 0 = No \| 1 = Yes \| 9 = Unknown  0 = No \| 1 = Yes | Categorical | Baseline  Both |

* Asher, John. transplantr: Audit and Research Functions for Transplantation. [Internet]. 2020. Available from: https://CRAN.R-project.org/package=transplantr

### Supplementary Table 3: Baseline characteristics of eligible individuals for an emulation of a target trial of ACEi/ARB versus no ACEi/ARB in individuals with myocardial infarction and preserved left ventricular ejection fraction, continued from Table 2

|  | **ACE I/ ARB** | **No ACE I/ ARB** | **Missing** | **SMD** | **SMD after IP weighting** |
| --- | --- | --- | --- | --- | --- |
| *During hospitalization* |  |  |  |  |  |
| Percutaneous coronary intervention | 9687 (90.6) | 3834 (81.1) | 0 | 0.275 | 0.034 |
| Angiography finding |  |  | 0.1 | 0.295 | 0.029 |
| Normal | 559 (5.2) | 634 (13.4) |  |  |  |
| One vessel | 5854 (54.8) | 2486 (52.6) |  |  |  |
| Two vessels | 2696 (25.2) | 979 (20.7) |  |  |  |
| Three vessels | 1267 (11.8) | 471 (10.0) |  |  |  |
| Left main | 316 (3.0) | 156 (3.3) |  |  |  |
| Stenosis class |  |  | 14 | 0.056 | 0.037 |
| A | 715 (7.5) | 338 (9.0) |  |  |  |
| B | 7026 (73.7) | 2699 (72.2) |  |  |  |
| C | 1793 (18.8) | 703 (18.8) |  |  |  |
| Proportion stenosis |  |  | 3.3 | 0.322 | 0.033 |
| 0% | 419 (4.0) | 498 (10.9) |  |  |  |
| <50% | 110 (1.1) | 109 (2.4) |  |  |  |
| 50-69% | 224 (2.2) | 154 (3.4) |  |  |  |
| 70-89% | 1471 (14.2) | 700 (15.3) |  |  |  |
| 90-99% | 3694 (35.7) | 1565 (34.3) |  |  |  |
| 100% | 4436 (42.8) | 1543 (33.8) |  |  |  |
| Intravenous beta blockers | 663 (6.2) | 237 (5.0) | 0 | 0.052 | 0.002 |
| Intravenous diuretics | 266 (2.5) | 68 (1.4) | 0 | 0.076 | 0.029 |
| Intravenous inotropic drugs | 146 (1.4) | 38 (0.8) | 0 | 0.054 | 0.004 |
| Intravenous nitrates | 744 (7.0) | 286 (6.1) | 0.1 | 0.037 | 0.017 |
| Other medications at baseline |  |  |  |  |  |
| Calcium channel blockers | 295 (2.8) | 128 (2.7) | 0 | 0.003 | 0.002 |
| Beta Blockers | 8995 (84.1) | 3647 (77.1) | 0 | 0.177 | 0.022 |
| Diuretics | 348 (3.3) | 149 (3.2) | 0 | 0.006 | 0.003 |
| Nitrates | 9128 (85.3) | 3963 (83.8) | 0 | 0.043 | 0.023 |
| Diabetes treatment | 79 (0.7) | 18 (0.4) | 0 | 0.048 | 0.021 |
| Year of index |  |  | 0 | 0.113 | 0.031 |
| 2010 | 316 (3.0) | 129 (2.7) |  |  |  |
| 2011 | 974 (9.1) | 468 (9.9) |  |  |  |
| 2012 | 1048 (9.8) | 462 (9.8) |  |  |  |
| 2013 | 964 (9.0) | 420 (8.9) |  |  |  |
| 2014 | 1029 (9.6) | 433 (9.2) |  |  |  |
| 2015 | 1054 (9.9) | 423 (8.9) |  |  |  |
| 2016 | 1049 (9.8) | 383 (8.1) |  |  |  |
| 2017 | 1071 (10.0) | 433 (9.2) |  |  |  |
| 2018 | 939 (8.8) | 487 (10.3) |  |  |  |
| 2019 | 981 (9.2) | 473 (10.0) |  |  |  |
| 2020 | 856 (8.0) | 375 (7.9) |  |  |  |
| 2021 | 416 (3.9) | 244 (5.2) |  |  |  |
| Hospital |  |  | 0 | 0.823 | 0.242 |
| Stockholm St Göran | 118 (1.1) | 263 (5.6) |  |  |  |
| Stockholm SÖS | 452 (4.2) | 240 (5.1) |  |  |  |
| Stockholm KI Solna | 116 (1.1) | 136 (2.9) |  |  |  |
| Stockholm KI Huddinge | 189 (1.8) | 240 (5.1) |  |  |  |
| Stockholm Danderyd | 333 (3.1) | 193 (4.1) |  |  |  |
| Uppsala | 184 (1.7) | 185 (3.9) |  |  |  |
| Eskiltuna | 326 (3.0) | 12 (0.3) |  |  |  |
| Linköping | 113 (1.1) | 149 (3.2) |  |  |  |
| Norrköping Vrinnevi | 128 (1.2) | 59 (1.2) |  |  |  |
| Motala | 52 (0.5) | 75 (1.6) |  |  |  |
| Jönköping | 106 (1.0) | 135 (2.9) |  |  |  |
| Eksjö | 88 (0.8) | 54 (1.1) |  |  |  |
| Ljungby | 43 (0.4) | 45 (1.0) |  |  |  |
| Kalmar | 322 (3.0) | 42 (0.9) |  |  |  |
| Kristianstad | 280 (2.6) | 14 (0.3) |  |  |  |
| Ängelholm | 105 (1.0) | 38 (0.8) |  |  |  |
| Malmö | 450 (4.2) | 91 (1.9) |  |  |  |
| Lund | 432 (4.0) | 76 (1.6) |  |  |  |
| Helsingborg | 292 (2.7) | 125 (2.6) |  |  |  |
| Halmstad | 310 (2.9) | 93 (2.0) |  |  |  |
| Varberg | 155 (1.4) | 126 (2.7) |  |  |  |
| Göteberg Sahlgrenska | 532 (5.0) | 131 (2.8) |  |  |  |
| Göteborg Östra | 238 (2.2) | 56 (1.2) |  |  |  |
| Göteborg Mölndal | 65 (0.6) | 83 (1.8) |  |  |  |
| Kungälv | 138 (1.3) | 54 (1.1) |  |  |  |
| Borås | 77 (0.7) | 79 (1.7) |  |  |  |
| Alingsås | 60 (0.6) | 38 (0.8) |  |  |  |
| Lidköping | 64 (0.6) | 66 (1.4) |  |  |  |
| Skövde | 232 (2.2) | 185 (3.9) |  |  |  |
| Karlstad | 270 (2.5) | 118 (2.5) |  |  |  |
| Örebro | 211 (2.0) | 67 (1.4) |  |  |  |
| Västerås | 282 (2.6) | 8 (0.2) |  |  |  |
| Köping | 136 (1.3) | 9 (0.2) |  |  |  |
| Falun | 275 (2.6) | 166 (3.5) |  |  |  |
| Mora | 82 (0.8) | 37 (0.8) |  |  |  |
| Sundsvall | 211 (2.0) | 61 (1.3) |  |  |  |
| Östersund | 156 (1.5) | 52 (1.1) |  |  |  |
| Umeå | 153 (1.4) | 43 (0.9) |  |  |  |
| Sunderbyn | 171 (1.6) | 17 (0.4) |  |  |  |
| Södertälje | 62 (0.6) | 79 (1.7) |  |  |  |
| Norrtälje | 40 (0.4) | 8 (0.2) |  |  |  |
| Enköping | 21 (0.2) | 24 (0.5) |  |  |  |
| Nyköping | 87 (0.8) | 7 (0.1) |  |  |  |
| Katrineholm | 107 (1.0) | 7 (0.1) |  |  |  |
| Värnamo | 68 (0.6) | 58 (1.2) |  |  |  |
| Växjö | 109 (1.0) | 67 (1.4) |  |  |  |
| Västervik | 98 (0.9) | 16 (0.3) |  |  |  |
| Oskarshamn | 108 (1.0) | 17 (0.4) |  |  |  |
| Visby | 43 (0.4) | 49 (1.0) |  |  |  |
| Karlskrona | 163 (1.5) | 77 (1.6) |  |  |  |
| Karlshamn | 29 (0.3) | 24 (0.5) |  |  |  |
| Hässleholm | 116 (1.1) | 16 (0.3) |  |  |  |
| Landskrona lasarett | 19 (0.2) | 9 (0.2) |  |  |  |
| Trelleborg | 122 (1.1) | 29 (0.6) |  |  |  |
| Ystad | 137 (1.3) | 73 (1.5) |  |  |  |
| Trollhättan | 458 (4.3) | 112 (2.4) |  |  |  |
| Arvika | 56 (0.5) | 26 (0.5) |  |  |  |
| Torsby | 67 (0.6) | 19 (0.4) |  |  |  |
| Karlskoga | 52 (0.5) | 17 (0.4) |  |  |  |
| Lindesberg | 60 (0.6) | 17 (0.4) |  |  |  |
| Avesta | 45 (0.4) | 27 (0.6) |  |  |  |
| Gävle | 182 (1.7) | 103 (2.2) |  |  |  |
| Bollnäs | 76 (0.7) | 44 (0.9) |  |  |  |
| Hudiksvall | 74 (0.7) | 23 (0.5) |  |  |  |
| Örnsköldsvik | 57 (0.5) | 22 (0.5) |  |  |  |
| Sollefteå | 43 (0.4) | <5 (0.1) |  |  |  |
| Skellefteå | 40 (0.4) | 23 (0.5) |  |  |  |
| Lycksele | 25 (0.2) | 14 (0.3) |  |  |  |
| Gällivare | 33 (0.3) | 5 (0.1) |  |  |  |
| Piteå | 60 (0.6) | 27 (0.6) |  |  |  |
| Kalix | 59 (0.6) | 15 (0.3) |  |  |  |
| Other | 34 (0.3) | 11 (0.2) |  |  |  |

### Supplementary Table 4 - Estimated 5-Year risks, and risk differences for the composite outcome for the intention-to-treat effect, stratified by subgroup

|  | **ACEi/ARB** | | | | | **No ACEi/ARB** | | | | |  | | |  | | | | |
| --- | --- | --- | --- | --- | --- | --- | --- | --- | --- | --- | --- | --- | --- | --- | --- | --- | --- | --- |
| **Subgroup^*^** | | **Events** | | **Risk, %**  **(95% CI)** | | | **Events** | | **Risk, %**  **(95% CI)** | | | **Risk ratio**  **(95% CI)** | | | **Risk difference, %**  **(95% CI)** | |  |  |
| 1. a) Male  b) Female | | | 579  150 | | 7.5 (6.9, 8.1)  8.8 (6.9, 11.6) | | | 240  87 | | 7.5 (6.2, 8.8)  8.9 (6.2, 12.2) | | | 1.01 (0.83, 1.22)  0.98 (0.66, 1.65) | | | 0.1 (-1.5, 1.4)  -0.2 (-4.0, 4.0) | |  |
| 1. a) STEMI  b) NSTEMI | | | 357  372 | | 8.3 (7.3, 9.2)  7.3 (6.6, 8.2) | | | 94  233 | | 9.2 (6.6, 12.2)  7.6 (6.4, 9.0) | | | 0.90 (0.68, 1.29)  0.96 (0.81, 1.16) | | | - 0.9 (-3.9, 1.8)  -0.3 (-1.6, 1.0) | |  |
| 1. a) Age <60 years   b) Age ≥ 60 years | | | 252  477 | | 5.9 (5.1, 6.5)  9.0 (8.3, 9.6) | | | 116  211 | | 5.6 (4.7, 6.8)  10.1 (8.7, 11.5) | | | 1.05 (0.85, 1.31)  0.89 (0.76, 1.02) | | | 0.3 (-1.1, 1.4)  -1.1 (-2.7, 0.1) | |  |

^*^ Confidence intervals estimated via bootstrapping with 200 samples. (STEMI – ST elevation myocardial infarction, NSTEMI, non ST elevation myocardial infarction)

### Supplementary Table 5 - Sensitivity analyses for intention-to-treat analysis with composite outcome

|  | | **ACEi/ARB** | | | | | **No ACEi/ARB** | | | | |  | | |  | | |
| --- | --- | --- | --- | --- | --- | --- | --- | --- | --- | --- | --- | --- | --- | --- | --- | --- | --- |
| **Sensitivity** ^*^ | **Events** | | **Risk, %**  **(95% CI)** | | **Events** | | | **Risk, %**  **(95% CI)** | | **Risk ratio  (95% CI)** | | | **Risk difference, %   (95% CI)** | | |  |  |
| 1. Restrict to coronary angiography showing obstructive coronary artery disease | | 693 | | 7.5 (6.9, 8.0) | | 289 | | | 8.1 (6.5, 9.3) | | 0.93 (0.79, 1.13) | | | -0.5 (-2.0, 0.9) | | |  |
| 1. Restricted to long term antithrombotic agents | | 728 | | 7.8 (7.0, 8.4) | | 327 | | | 8.1 (7.1, 9.4) | | 0.96 (0.78, 1.10) | | | -0.4 (-0.2, 0.8) | | |  |
| 1. Restrict recruitment prior to the COVID-19 pandemic (<2020) | | 700 | | 8.0 (7.3, 8.7) | | 314 | | | 8.4 (7.3, 9.7) | | 0.95 (0.80, 1.12) | | | -0.4 (-1.9, 0.9) | | |  |
| 1. a) Complete case analysis | | 450 | | 6.9 (6.3, 7.6) | | 175 | | | 7.5 (6.0, 9.0) | | 0.93 (0.75, 1.19) | | | -0.6 (-2.2, 1.1) | | |  |
| b) Impute the median for continuous variables | | 729 | | 7.7 (7.1, 8.4) | | 327 | | | 8.2 (7.0, 9.4) | | 0.94 (0.80, 1.12) | | | -0.5 (-1.8, 0.8) | | |  |
| 1. Baseline covariates included in pooled logistic regression model | | 729 | | 7.3 (6.8, 7.9) | | 327 | | | 7.7 (6.8, 8.7) | | 0.95 (0.81, 1.09) | | | -0.4 (-1.7, 0.6) | | |  |
| 1. Adjustment for age and sex only | | 729 | | 7.6 (7.1, 8.2) | | 327 | | | 8.1 (7.3, 8.9) | | 0.94 (0.85, 1.05) | | | -0.5 (-1.3, 0.3) | | |  |
| 1. Truncation of inverse probability weights | | 729 | | 7.7 (7.1, 8.4) | | 327 | | | 8.4 (7.3, 9.4) | | 0.91 (0.79, 1.08) | | | -0.7 (-1.9, 0.6) | | |  |
| 1. Identify day of eligibility, and then clone and censor ^b^ | | 705 | | 8.0 | | 484 | | | 9.0 | | 0.89 | | | -1.0 | | |  |

^*^ Confidence intervals estimated via bootstrapping with 200 samples. ^b^ No confidence intervals estimated via bootstrapping due to computational restrictions.

### Supplementary Table 6 - Sensitivity analyses for intention-to-treat analysis with heart failure and myocardial infarction outcomes

|  | | **ACEi/ARB** | | | **No ACEi/ARB** | | |  | |  | |
| --- | --- | --- | --- | --- | --- | --- | --- | --- | --- | --- | --- |
| **Sensitivity^*^** | **Events** | | **Risk, %**  **(95% CI)** | **Events** | | **Risk, %**  **(95% CI)** | **Risk ratio,   (95% CI)** | | **Risk difference, %,  (95% CI)** | |  |
| 1. Censoring at death for the outcome of myocardial infarction | 392 | | 4.2 (3.8, 4.7) | 229 | | 4.6 (3.8, 5.4) | 0.92 (0.75, 1.13) | | -0.4 (-1.3, 0.5) | |  |
| 1. Censoring at death for the outcome of heart failure | 123 | | 1.4 (1.1, 1.7) | 43 | | 1.2 (0.8, 1.9) | 1.12 (0.72, 1.85) | | 0.2 (-0.4, 0.6) | |  |

^*^ Confidence intervals calculated estimated via bootstrapping with 200 samples

### Supplementary Table 7 - Sensitivity analyses for per-protocol analysis with composite outcome

|  | **ACEi/ARB** | | | | **No ACEi/ARB** | | |  | |  | |
| --- | --- | --- | --- | --- | --- | --- | --- | --- | --- | --- | --- |
| **Sensitivity^*^** | | **Events** | **Risk, %**  **(95% CI)** | **Events** | | **Risk, %**  **(95% CI)** | **Risk ratio,   (95% CI)** | | **Risk difference, %,  (95% CI)** | |  |
| (1) Adjustment for age and sex only | | 509 | 6.5 (5.9, 7.1) | 229 | | 6.7 (5.9, 7.5) | 0.97 (0.84, 1.12) | | -0.2 (-1.2, 0.7) | |  |
| (2a) Treatment length calculated as one pill per day with 180 day grace period | | 567 | 6.8 (6.2, 7.5) | 229 | | 6.7 (5.7, 8.0) | 1.02 (0.85, 1.25) | | - 1. (-1.2, 1.4) | |  |
| (2b) Fixed 90 day treatment length from each dispensation with 90 day grace period | | 479 | 6.4 (5.8, 7.2) | 229 | | 6.7 (5.6, 8.0) | 0.96 (0.78, 1.19) | | -0.3 (-1.7, 1.0) | |  |

^*^ Confidence intervals calculated estimated via bootstrapping with 200 samples

### Supplementary Table 8 - Sensitivity analyses for per-protocol analysis with heart failure outcome

|  | **ACEi/ARB** | | | **No ACEi/ARB** | | |  | |  | |
| --- | --- | --- | --- | --- | --- | --- | --- | --- | --- | --- |
| **Sensitivity^*^** | **Events** | **Risk, %**  **(95% CI)** | **Events** | | **Risk, %**  **(95% CI)** | **Risk ratio,   (95% CI)** | | **Risk difference, %,  (95% CI)** | |  |
| Decreasing diagnostic time of heart failure by six months | 59 | 1.4 (1.1, 1.7) | 100 | | 1.0 (0.6, 1.6) | 1.37 (0.90, 2.29) | | 0.4 (-0.1, 0.9) | |  |

^*^ Confidence intervals calculated estimated via bootstrapping with 200 samples. Individuals with a heart failure diagnosis occurring in the first six months were set to month 1

### Supplementary Figure 1 - Estimated risk of the composite outcome, death, myocardial infarction (MI) and heart failure (HF) and in those assigned ACEi/ARB versus no ACEi/ARB in the per-protocol analysis^*^


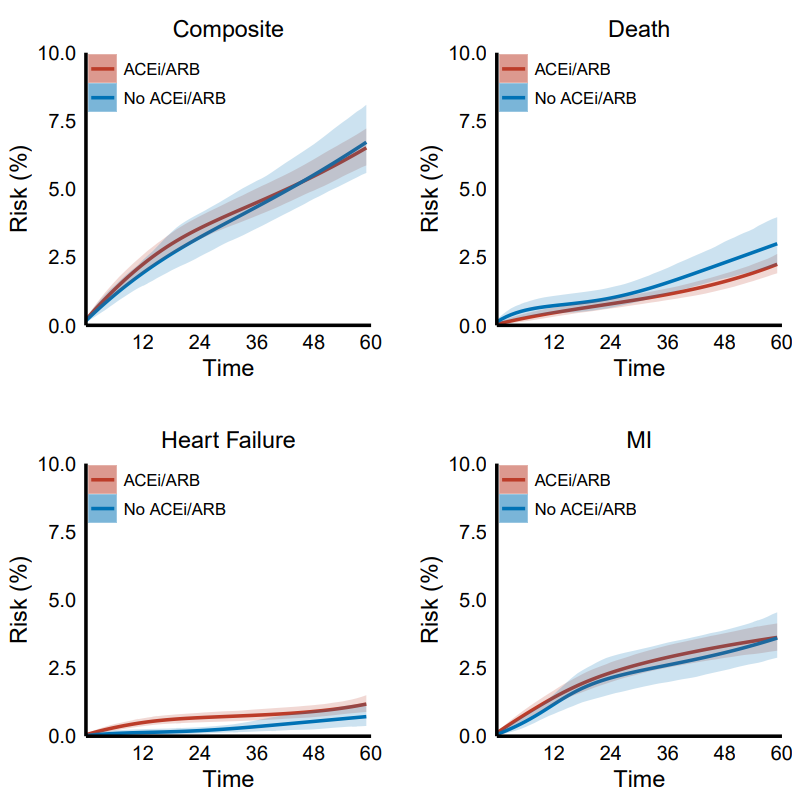


^*^ Shaded intervals represent limits of the pointwise 95% CIs.
